# 本科仪器分析实验设计与实践：固相微萃取-液相色谱检测环境水体中的多环芳烃

**DOI:** 10.3724/SP.J.1123.2024.11024

**Published:** 2025-10-08

**Authors:** Jun LIU, Jiangyi WU, Jintao LI, Nengwang CHEN, Xiaojia HUANG

**Affiliations:** 1.厦门大学环境与生态学院，福建 厦门 361000; 1. College of the Environment & Ecology，Xiamen University，Xiamen 361000，China; 2.厦门大学嘉庚学院，福建 漳州 363000; 2. Xiamen University Tan Kah Kee College，Zhangzhou 363000，China

**Keywords:** 本科实验教学, 固相微萃取, 仪器分析, 多环芳烃, 液相色谱, undergraduate experimental teaching, solid-phase microextraction （SPME）, instrumental analysis, polycyclic aromatic hydrocarbons （PAHs）, liquid chromatography （LC）

## Abstract

从环境科学专业特点出发，结合科研课题，设计了一项可用于本科教学的仪器分析开放型实验。该实验根据多环芳烃（PAHs）分子结构特点，以9-乙烯基蒽和苯乙烯为混合功能单体，利用“原位”聚合技术合成基于整体材料的固相微萃取（SPME）纤维束，并将其作为萃取介质，在优化条件下，结合高效液相色谱法（HPLC）检测环境水样中的PAHs。本实验设计并构建了一个完整的仪器分析实验流程，具体主要包括SPME纤维束的制备和表征、萃取实验条件优化、实际水样前处理和HPLC分析检测等。在教师引导下，学生开展实验前期相关知识学习、实验中期提升实验操作技能、实验后期分析数据并撰写研究报告。通过该课程全流程学习，不仅激发了学生的学习兴趣，还可培养其科学思维，提高分析和解决问题的能力，为培养新时代环境科学专业高素质人才奠定基础。

## Abstract (en)

A research-project-based open instrumental-analysis experiment was designed for undergraduate teaching led by the attributes of an environmental science major. In this experiment， porous monolithic fibers are *in-situ* synthesized from 9-vinylanthracene and styrene （as a monomer mixture） based on the molecular characteristics of studied polycyclic aromatic hydrocarbons （PAHs）， and then used as an extractant for solid-phase microextraction （SPME）. A method for reliably monitoring PAHs in environmental water samples was developed by combining SPME with high performance liquid chromatography （HPLC）. Students designed and constructed a complete experimental instrumental-analysis process， which included preparing and characterizing the monolith fibers， optimizing the SPME conditions， preparing environmental water samples， and analysis by HPLC. Guided by teachers， undergraduates learned relevant knowledge prior to performing the experiment， improved their operating skills during the experiment， analyzed data， and wrote research reports during the latter stage of the course. Undergraduates' interest is stimulated by completing the experiment， while scientific thinking， analysis， and problem-solving abilities are concurrently improved and cultivated. The present course also lays the foundation for cultivating future high-quality environmental-science talents.

现代仪器分析技术不仅是环境监测领域的重要分析测试方法，而且是科学研究强有力的支撑手段^［1，2］^，因此熟悉并掌握各类现代仪器分析原理和实验技术是培养环境高素质人才的重要环节。仪器分析理论和实验课程是现代仪器分析原理和实验技术的主要教学渠道，传统的仪器分析实验教学侧重于仪器的原理、结构及操作步骤，多为单一验证性实验，缺乏实际应用型实验案例，不利于学生综合素质和创新思维的培养^［3］^。仪器分析实验设计应从授课对象的专业特点出发，与科研成果紧密结合，让学生体验本专业的科研实践，才能有效提高人才培养效果和质量^［4-7］^。对于环境科学专业来说，仪器分析与环境样品前处理技术相结合，可以使学生充分了解样品前处理的重要性^［8，9］^，让学生得到完整的样品分析流程的训练，培养其分析和解决实际问题的能力，从而提高其专业素质、科学思维和综合能力。

多环芳烃（PAHs）是由两个或两个以上苯环组成的一类芳香族化合物，也是全球环境污染问题中环境监测领域关注的焦点之一^［10，11］^。PAHs广泛存在于环境中，且水体、土壤和空气中的PAHs通常以混合物形式存在，最终通过食物链进入人体，具有明显的致癌、致畸、致突变危害^［12］^，因此检测水体中的多环芳烃具有重要的现实意义。高效液相色谱法（HPLC）因其优越的分离性能和性价比而成为分析环境中有机污染物的主要手段^［13］^。由于环境样品中的PAHs含量极低且基质复杂，因此需进行合适的样品前处理以消除样品基底对分析结果的干扰和提高检测灵敏度，同时减少杂质对仪器的损害，使样品的状态和性质符合仪器的工作条件以保护分析仪器。传统样品前处理技术，如索式提取法、液液萃取法、超声波萃取法、固相萃取法等虽已被用于PAHs的富集，但存在操作繁琐、溶剂消耗量大、分析时间长等缺点，而新兴的前处理技术如固相微萃取（SPME）具有操作简便、溶剂消耗少和环境友好等特点已被广泛使用^［14-17］^。SPME是20世纪90年代兴起的样品前处理技术，主要的形式为纤维式、管式、薄膜式、搅拌棒式等，其中，纤维式SPME技术由于操作简便和良好的萃取性能，目前在环境样品分析中的应用最为广泛^［18］^。在SPME技术中，其核心是涂层，通常根据“相似相溶”的原理来选择合适的SPME涂层以提高富集性能。

本实验在前期课题研究^［19-22］^基础上，将SPME与液相色谱-二极管阵列检测器（HPLC-DAD）相结合的实验技术引入环境科学专业的本科仪器分析实验教学中，让学生自制基于整体材料的SPME纤维束并用于萃取和富集环境水样中的PAHs，然后在优化的萃取条件下，结合HPLC进行定性定量分析。该实验设计将科研成果转化为教学实践，旨在激发学生对污染物监测的兴趣，增强学生的实验操作技能，培养学生的科学思维，使学生充分意识到前处理对环境监测的重要性以及仪器分析的重要意义，为培养新时代符合国家和社会需求的高素质人才奠定基础。

## 1 实验部分

### 1.1 仪器与试剂

U3000高效液相色谱仪（配二极管阵列检测器，Thermo，美国），XL 30型扫描电镜（SEM，Philips，荷兰），IRAffinity-1S傅里叶变换红外光谱仪（FT-IR，Shimadzu，日本），CP114型分析天平（Ohaus，美国），KQ-100型超声波清洗仪（昆山市超声仪器有限公司），JBZ-14型磁力搅拌器（上海康仪仪器有限公司）。

萘（naphthalene，Nap）、芴（fluorene，Flu）、苊（acenaphthene，Ana）、菲（phenanthrene，Phe）、荧蒽（fluoranthene，Flt）、芘（pyrene，Pyr）和䓛（chrysene，Chr）标准品均购自阿拉丁试剂公司；甲醇、乙腈均为色谱纯，购自美国Tedia试剂公司；苯乙烯（99%）、9-乙烯基蒽（97%）、偶氮二异丁腈（AIBN，97%）购自上海麦克林生化科技有限公司；二甲基亚砜（DMSO，≥99.5%）购自上海西陇化工有限公司；乙二醇二甲基丙烯酸酯（EDMA，97%）购自天津Alfa Aesar试剂公司；实验用水均为Milli-Q超纯水（18.2 MΩ·cm）。

### 1.2 色谱分析条件

Kromasil 100-5 C18色谱柱（250 mm×4.6 mm，5 µm）；流动相为（A）超纯水和（B）乙腈，流速为1.0 mL/min，进样量20 μL。梯度洗脱程序如下：0~18 min，73%B；18~19 min，73%B~83%B；19~25 min，83%B；25~26 min，83%B~73%B。7种PAHs的检测波长：Nap、Ana为220 nm，Flt、Pyr为240 nm，Phe为250 nm，Flu、Chr为260 nm。

### 1.3 实验步骤

#### 1.3.1 湖水样品预处理

采集校园内芙蓉湖的湖水样品。水样采集后，用0.45 μm水系微孔滤膜过滤，过滤后样品于4 ℃冰箱储存，备用。

#### 1.3.2 SPME纤维束的制备

首先称取22 mg 9-乙烯基蒽和44 mg苯乙烯，置于小烧杯中混合均匀，作为混合功能单体。然后称取81 mg交联剂EDMA、150 mg致孔剂DMSO和3.0 mg引发剂AIBN，依次加入装有混合功能单体的小烧杯中，混合均匀，放入超声波清洗仪超声至固体完全溶解，将超声后的混合溶液注入长度为10 cm、内径为530 μm的空心玻璃毛细管模具中，然后利用硅胶片将毛细管两端密闭，在70 ℃下加热12 h进行原位聚合反应，如图1所示。反应完成后去除2.0 cm长的毛细管，暴露出合成好的整体材料，用甲醇和超纯水依次浸泡整体材料以除去致孔剂和未反应的组分，即得到半透明、白色的细整体纤维，之后将4根成分相同的整体纤维捆绑在一起后组成SPME纤维束。如图2所示，SPME纤维束中半透明、白色的为整体材料部分。

**图1:**
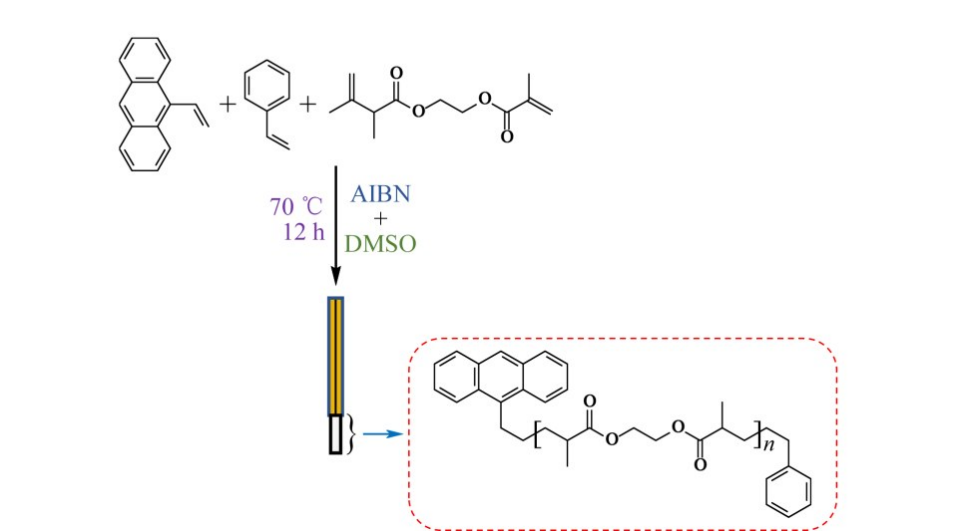
原位聚合反应示意图 AIBN： azobisisobutyronitrile； DMSO： dimethyl sulfoxide.

**图2:**
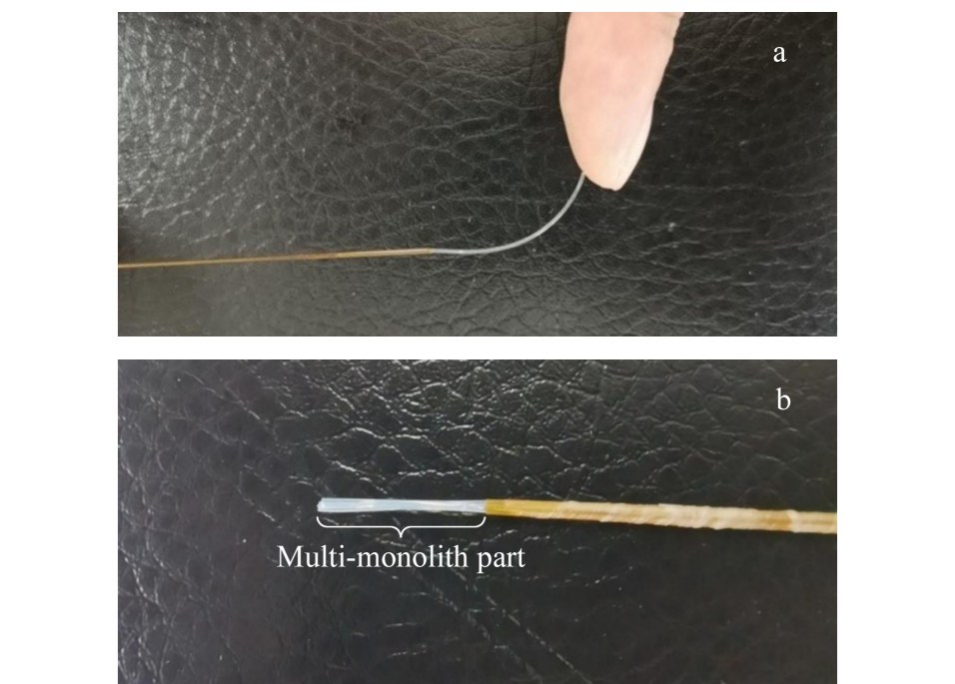
自制（a）单根整体纤维和（b）纤维束实物图

#### 1.3.3 SPME过程

吸附前，先用超纯水彻底冲洗SPME纤维束。然后，取20 mL样品溶液于萃取瓶中，将SPME纤维束的整体材料部分插入样品溶液中进行吸附萃取50 min，溶液磁力搅拌，搅拌速度为300 r/min。萃取完成后，将SPME纤维束取出并插入含有400 μL乙腈的玻璃小管中，解吸20 min，解吸过程同样进行磁力搅拌，搅拌速度为300 r/min。解吸完成后，解吸液用0.22 μm聚四氟乙烯滤膜过滤后进行HPLC分析。

## 2 结果与讨论

### 2.1 自制SPME纤维束的表征

学生利用FT-IR光谱仪对自制纤维束的吸附材料进行分子结构表征，结果如图3所示。FT-IR谱图中，2 952 cm^-1^和1 730 cm^-1^处的特征吸收峰分别为合成整体材料分子结构中的烷基和羰基伸缩振动吸收峰；1 602、1 456和1 386 cm^-1^处的吸收峰则证实材料中苯环的存在。

**图3:**
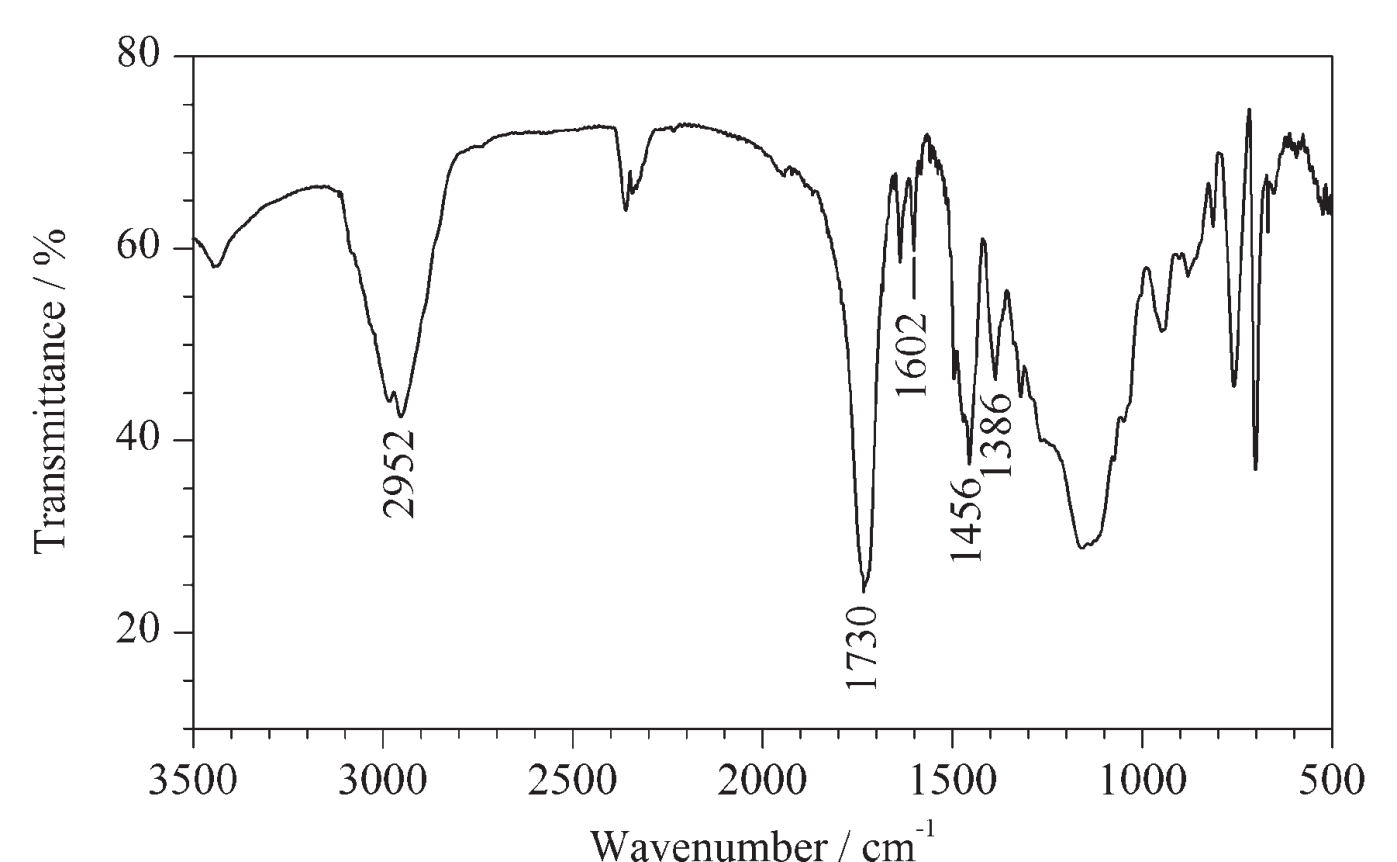
自制整体纤维的FT-IR谱图

同时利用SEM技术观察整体材料的微观形貌。由图4a（34×放大倍率）可以看出，合成的聚合物细纤维表面连成一体且光滑。由放大20 000倍的图像（见图4b）中可以看出，所制备的整体材料具有较好的致密性，但存在一定空隙，这有利于传质过程。扫描电镜实验由教师完成，扫描结果向学生展示，学生在实验报告中对其合成的整体材料的微观形貌进行阐述和讨论。

**图4:**
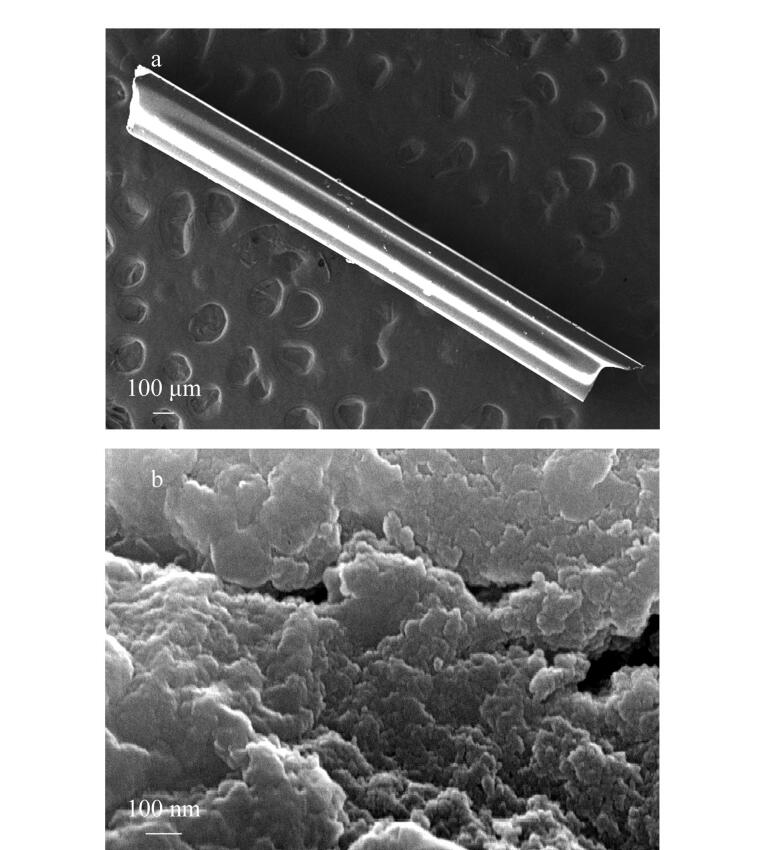
整体纤维放大（a）34倍和（b）20 000倍的SEM图

### 2.2 萃取条件的优化

为得到最佳的萃取参数，本实验采用单因素分析法对影响吸附效果的吸附时间、解吸时间、离子强度等参数进行了优化。优化过程中采用的PAHs标准溶液的质量浓度为10 μg/L，通过色谱峰的峰面积评估SPME纤维束对PAHs的萃取效率。

#### 2.2.1 吸附时间

吸附时间是影响萃取效率的重要因素，足够的吸附时间可以使样品溶液中的目标物与吸附材料充分作用，从而提高萃取效率。本实验首先考察了吸附时间在30~70 min范围内SPME纤维束萃取效率的变化。如图5所示，当吸附时间从30 min增加到50 min时，SPME纤维束的萃取效率明显增加，并在吸附时间为50 min时达到最佳的萃取效果，继续增加吸附时间，萃取效率变化不显著。因此，选择50 min为最优吸附时间。

**图5:**
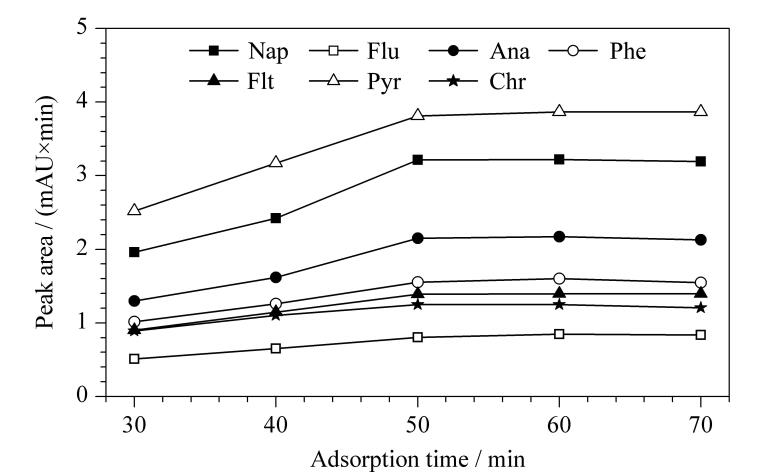
吸附时间对PAHs萃取性能的影响 Nap： naphthalene； Flu： fluorene； Ana： acenaphthene； Phe： phenanthrene； Flt： fluoranthene； Pyr： pyrene； Chr： chrysene.

#### 2.2.2 解吸时间

解吸时间影响纤维束上吸附的PAHs能否被完全洗脱。较短的解吸时间会影响目标物的洗脱，存在残留效应。为了达到最佳的洗脱效率，本实验在15~30 min范围内考察了解吸时间的影响。如图6可知，随着解吸时间的增加，解吸过程逐渐达到平衡，在20 min时洗脱效率达到最大。因此，在后续实验中设置解吸时间为20 min。

**图6:**
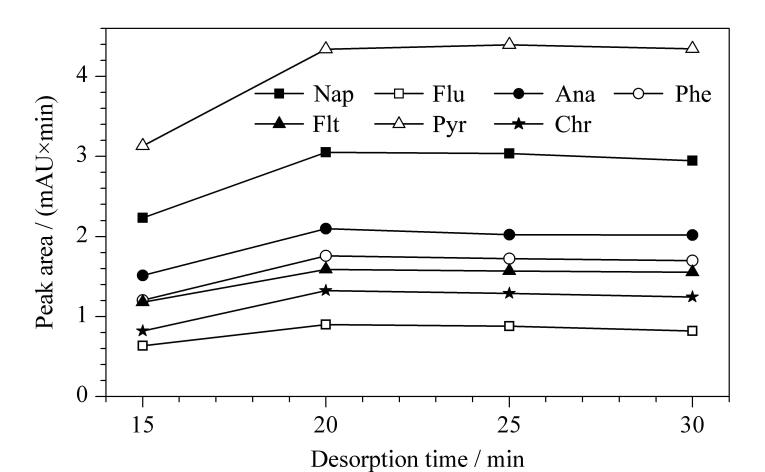
解吸时间对PAHs洗脱性能的影响

#### 2.2.3 离子强度

样品溶液中的离子强度会影响PAHs在吸附剂和溶液间的分配系数，离子强度的增加通常可提高萃取效率，但过高的离子强度会增加溶液黏稠度，影响扩散吸附，从而影响SPME纤维束的萃取性能。本实验在50 mL超纯水中加入0、2.5、5.0、7.5和10 g的NaCl，考察离子强度对SPME纤维束萃取PAHs的影响。如图7所示，随着NaCl加入量的增加，SPME纤维束对目标物的萃取效率显著降低。根据实验结果，在后续实验中不加入NaCl。

**图7:**
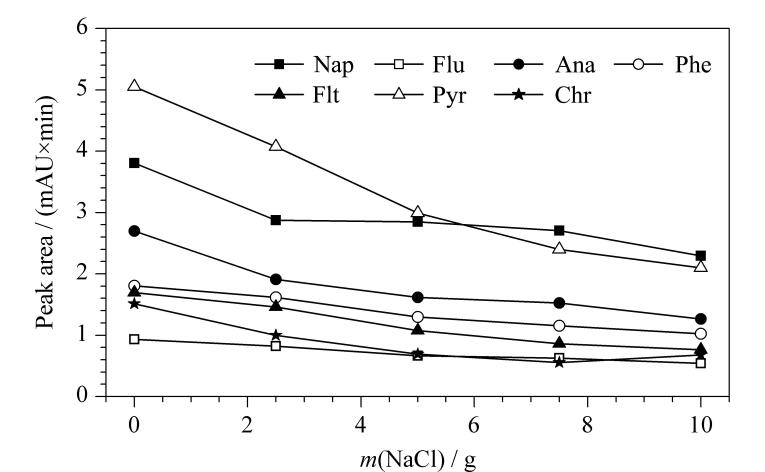
离子强度对PAHs萃取性能的影响

### 2.3 线性范围、检出限和定量限

在最优萃取条件下，考察了所建立的SPME-HPLC-DAD分析方法的性能。配制一系列不同质量浓度（0.20、0.50、1.0、5.0、10、20、50和100 μg/L）的标准溶液，然后利用所建立的SPME-HPLC-DAD方法进行萃取和测定，获得方法的标准工作曲线、线性范围和相关系数（*R* ^2^）。如表1所示，Chr的线性范围为1.0~100 μg/L，其余6种PAHs的线性范围为0.20~100 μg/L。所有工作曲线具有良好的线性相关系数，*R* ^2^为0.990 1~0.997 4。检出限（LOD）和定量限（LOQ）分别通过计算3倍和10倍的色谱峰信噪比（*S/N*）获得。表1结果表明，所建方法的LOD和LOQ分别为0.021~0.089 μg/L和0.070~0.30 μg/L。

**表1:** 7种PAHs的线性范围、相关系数、富集倍数、检出限、定量限和精密度

Compound	Linear range/（μg/L）	*R* ^2^	EF	LOD/（μg/L）	LOQ/（μg/L）	RSDs/% （*n*=5）	
						Intra-day	Inter-day
Nap	0.20‒100	0.9974	94	0.039	0.13	4.1	1.4
Flu	0.20‒100	0.9916	113	0.048	0.16	6.2	2.3
Ana	0.20‒100	0.9920	126	0.048	0.16	4.6	1.4
Phe	0.20‒100	0.9918	106	0.032	0.11	5.3	3.1
Flt	0.20‒100	0.9960	109	0.021	0.070	2.2	2.0
Pyr	0.20‒100	0.9901	61	0.049	0.16	5.2	4.2
Chr	1.0‒100	0.9920	84	0.089	0.30	8.3	3.9

富集倍数（EF）是衡量SPME技术对目标化合物富集能力的一个重要指标，其计算公式如式（1）所示：

EF=*C*_1_/*C*_0 _ （1）

其中，*C*_0_为样品溶液中PAHs的初始质量浓度（μg/L），*C*_1_为解吸液中PAHs的质量浓度（μg/L）。本实验中7种PAHs的富集倍数为61~126，表明所建立的方法能有效富集痕量PAHs。

对加标水平为10 μg/L的PAHs标准溶液进行检测，同一SPME纤维束在同一天内连续测定5次的日内精密度（RSD）为2.2%~8.3%，同一SPME纤维束连续测定5天的日间精密度为1.4%~4.2%，结果表明所建立的测定方法具有良好的实验重复性。

### 2.4 水样的测定和加标回收率

利用所建方法，对校园湖水中的PAHs进行富集与检测，并进行3个水平的加标回收率试验，计算回收率和相对标准偏差，测定的结果如表2所示。在校园湖水中未检测到PAHs，不同水平下的加标回收率为79.5%~111%（RSD=1.1%~8.8%），说明所建方法具有良好的准确性和抗干扰能力。图8为湖水样品和加标水平为10 μg/L的湖水样品经SPME后的典型色谱图，可以看出，经过前处理后，未见有干扰物质影响目标物的定性和定量分析。以上研究结果表明本实验建立的SPME-HPLC-DAD方法适用于环境水体中痕量PAHs的检测。

**表2:** 湖水样品中7种多环芳烃在3个水平下的加标回收率（*n*=5）

Compound	1.0 μg/L		10 μg/L		50 μg/L	
	Recovery/%	RSD/%	Recovery/%	RSD/%	Recovery/%	RSD/%
Nap	80.4	8.5	93.9	3.4	97.7	3.4
Flu	90.5	2.0	93.0	2.2	101	2.2
Ana	97.6	6.1	103	4.4	111	4.4
Phe	79.5	2.9	85.2	3.0	99.4	3.0
Flt	104	1.1	104	3.5	102	3.5
Pyr	96.1	8.8	104	3.6	101	3.6
Chr	99.5	7.4	91.2	8.0	84.1	1.2

**图8:**
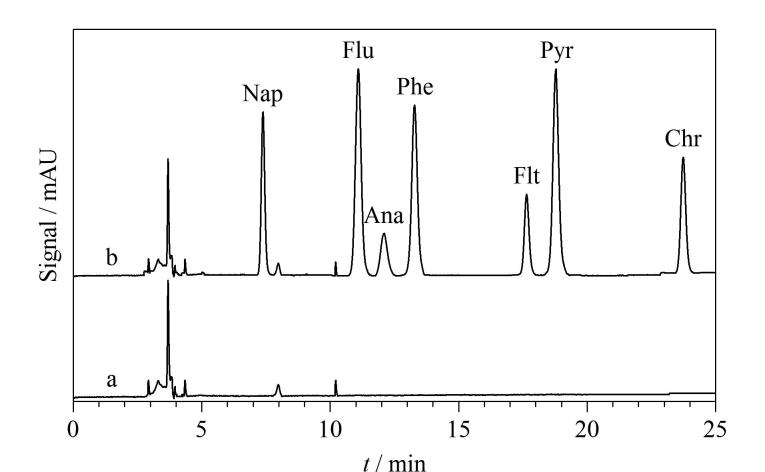
（a）实际湖水样品和（b）加标（10 μg/L）湖水样品经SPME后的色谱图

## 3 实验的组织与教学安排

本实验为环境科学专业仪器分析实验课程的开放型实验项目，学生3~4人为一组，共同设计实验方案并分工协作完成实验。该开放型实验的开展分为3个阶段，需5周时间来完成，实验教学内容安排如图9所示。实验前期为第一阶段，共2周，让学生通过阅读实验相关的文献，了解纤维束SPME原理和整体纤维制备方法、材料表征手段、HPLC定性定量原理、用于PAHs检测的主要技术，在此基础上，完成实验方案的设计。实验中期为第二阶段，约2周，学生利用课外及周末的时间来完成实验部分的内容。在该阶段实际操作中，指导学生制备基于整体材料的SPME纤维束，并用FT-IR和SEM进行表征；同时让学生对自制的纤维束进行萃取和解析条件的优化，建立SPME-HPLC-DAD测定PAHs的方法。在实际应用中，指导学生测定实际水样中PAHs含量，进行加标回收试验。实验后期为第三阶段，共1周，学生需对实验数据进行整理、分析和讨论，每个实验小组提交一份综合性实验研究报告。

**图9:**
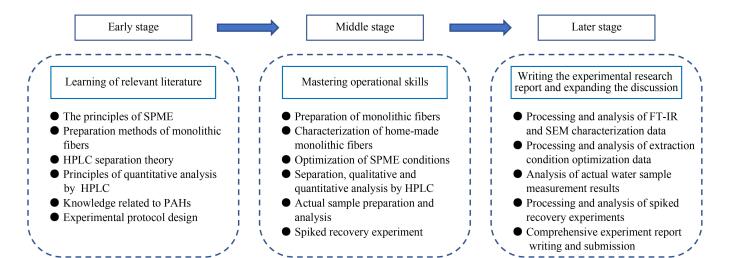
仪器分析实验教学安排

## 4 教学反思及注意事项

（1）本实验以探索型实验教学模式开展，学生全过程参与文献阅读、纤维束的制备和红外表征、仪器分析方法的建立、实际水样的测定以及最后的数据整理和报告撰写，全过程注重在教师的引导作用下激发学生的科研兴趣。教师在实验开始前应先引导学生阅读与实验密切相关且高质量的文献资料，让学生能迅速把握实验重点与难点；学生在实验方案设计过程中，教师给予把关指导，以确保实验方案的可行性；在实验过程中，指导教师与学生一起探讨每一个实验环节得到的实验数据，分析这些数据能说明什么问题、得出怎样的结论，评估实验结果是否符合预期，如不符合，分析可能的原因以及补充完善相关实验。在这个过程中，可以逐步培养学生分析数据、逻辑推理及对实验结果进行科学解释的能力。（2）整体纤维束的制备需花费较多的时间，学生往往未能一次性成功制备出理想的纤维束，因此制备的步骤需由教师或助教指导培训后完成，为节省时间也可由教师或助教完成，以视频或照片的方式给学生演示纤维束制备的过程。（3）通过实验，让学生掌握整体纤维束在解吸实验之后需要进一步用甲醇和超纯水进行残留物的清洗和活化，以对纤维束进行重复使用，降低分析成本。（4）学生对前沿的SPME样品前处理技术和功能材料的应用表现出极大的兴趣，基于科研课题研究的基础，后续可以拓展其他吸附材料作为环境有机污染物的监测，并应用到本科实验教学中。（5）实验过程中应牢记安全第一。学生在实验安全教育和培训后再进行实验，大型仪器也需要在培训熟练的情况下按操作步骤进行使用，同时要求教师或助教指导。以上从教学实践中获得的经验和注意事项将有助于提高实验教学质量和效果，同时也有利于提高学生的实验素质和综合能力。

## 5 结语

本实验在原有的仪器分析实验项目上进行扩展，增加基于整体材料的SPME前处理技术，并且在教学过程中引入了红外光谱和电镜扫描表征技术。在教师指导下，学生对实验方案进行优化并实施，同时利用自制的整体纤维束完成环境水样中PAHs的萃取和富集，再利用HPLC进行分析检测。实验过程涉及一系列基础实验操作，可以强化学生的动手实践能力；FT-IR的表征、色谱分析测定在校区仪器分析实验教学中心的实验室进行，学生在教师指导下反复进行多次实验，能够达到熟练操作的程度，并在一定程度上增强了对现代仪器分析原理和实验技术的掌握。该实验设计不仅丰富了传统仪器分析实验项目的内容，同时拓展了学生的知识面和视野，使学生不仅能够领会功能材料的性质及应用，也对HPLC定性定量分析有了更全面和深刻的理解。通过对有机污染物的富集和检测，激发了学生对环境监测和环境保护的热情，增强了其责任感和使命感。通过全程参加整个实验，学生得到较为完整的科研训练，他们先进行了实验前期知识的储备，随着实验推进，在实验中期及后期又实现了知识的有效应用，并且自身能力也得到了显著提升。该实验改变了原来传统的验证实验模式，使之更加适应新时代实验教学发展趋势，更有利于学生科学思维的养成和综合能力的提升，也更有助于新时代高素质人才的培养。
